# Reconstruction of the experimentally supported human protein interactome: what can we learn?

**DOI:** 10.1186/1752-0509-7-96

**Published:** 2013-10-02

**Authors:** Maria I Klapa, Kalliopi Tsafou, Evangelos Theodoridis, Athanasios Tsakalidis, Nicholas K Moschonas

**Affiliations:** 1 Metabolic Engineering and Systems Biology Laboratory, Institute of Chemical Engineering Sciences, Foundation for Research and Technology-Hellas (FORTH/ICE-HT), Rio, Patras, Greece; 2 Department of General Biology, School of Medicine, University of Patras, Rio, Patras, Greece; 3 Computer Engineering and Informatics Department, University of Patras, Rio, Patras, Greece

**Keywords:** Human protein interactome analysis, Human protein-protein interaction (PPI) databases, Network biology, PPI network reconstruction

## Abstract

**Background:**

Understanding the topology and dynamics of the human protein-protein interaction (PPI) network will significantly contribute to biomedical research, therefore its systematic reconstruction is required. Several meta-databases integrate source PPI datasets, but the protein node sets of their networks vary depending on the PPI data combined. Due to this inherent heterogeneity, the way in which the human PPI network expands via multiple dataset integration has not been comprehensively analyzed. We aim at assembling the human interactome in a global structured way and exploring it to gain insights of biological relevance.

**Results:**

First, we defined the UniProtKB manually reviewed human “complete” proteome as the reference protein-node set and then we mined five major source PPI datasets for direct PPIs exclusively between the reference proteins. We updated the protein and publication identifiers and normalized all PPIs to the UniProt identifier level. The reconstructed interactome covers approximately 60% of the human proteome and has a scale-free structure. No apparent differentiating gene functional classification characteristics were identified for the unrepresented proteins. The source dataset integration augments the network mainly in PPIs. Polyubiquitin emerged as the highest-degree node, but the inclusion of most of its identified PPIs may be reconsidered. The high number (>300) of connections of the subsequent fifteen proteins correlates well with their essential biological role. According to the power-law network structure, the unrepresented proteins should mainly have up to four connections with equally poorly-connected interactors.

**Conclusions:**

Reconstructing the human interactome based on the *a priori* definition of the protein nodes enabled us to identify the currently included part of the human “complete” proteome, and discuss the role of the proteins within the network topology with respect to their function. As the network expansion has to comply with the scale-free theory, we suggest that the core of the human interactome has essentially emerged. Thus, it could be employed in systems biology and biomedical research, despite the considerable number of currently unrepresented proteins. The latter are probably involved in specialized physiological conditions, justifying the scarcity of related PPI information, and their identification can assist in designing relevant functional experiments and targeted text mining algorithms.

## Background

Deciphering the structure and dynamics of the protein-protein interaction (PPI) networks is among the major objectives of the systems biology research in the quest for the mechanisms of life. For the human protein interactome in particular, its reconstruction and further exploration of its topology and dynamics are expected to have a significant impact in biomedical research and applications [1,2]. The number of experimentally supported PPIs has drastically increased for model organisms since 2000 [3-7] and for the human interactome since 2005 [8,9] mainly due to the gradually increasing number of high-throughput methodologies for PPI detection. The experimentally identified PPIs are mined from the literature and stored in bulk in PPI databases, most of which are repositories for many species. For the human interactome, the various source PPI databases report the protein identifiers at different molecular levels of biological information, and include protein interaction sets of limited overlap due to own literature mining criteria, differences in PPI incorporation rates from small-scale experiments, as well as differences in methods for PPI selection, curation and updating [10-14]. Therefore, several PPI meta-databases also exist, combining information from multiple source databases [15-23]. However, as each meta-database has distinct curation objectives and methods for data normalization and integration, the use of its combined PPI dataset may not be straight away comparable to the direct query on the source databases [11,12]. In addition, it is worth mentioning that the set of protein nodes of a meta-database network varies depending on the PPIs of the employed source datasets, and it may change upon updating or incorporation of new datasets. This fact creates heterogeneity between the various PPI meta-databases and hinders the direct comparison among their networks [11]. Because of this inherent heterogeneity, although there have been many studies comparing a variety of PPI datasets [10-14], the way in which the human protein interactome expands via the integration of multiple datasets has not been comprehensively explored; therefore, a global perspective of the biology emerging from the network structure is still eluding.

The objective of the present study is to reconstruct the current experimentally supported network of direct human protein interactions in a global structured way, explore it to obtain information about the fraction of the human proteome that it currently involves, discuss the biological role of proteins within the topology of the network, and identify the presently absent from the network (“orphan”) proteins. To this end, we started by defining the UniProtKB manually reviewed human “complete” proteome [24] as the reference set of nodes that the human PPI network can have. Then, we mined five major source PPI databases, i.e.: HPRD [25], IntAct [26], MINT [27], DIP [28] and BioGRID [29], for direct interactions exclusively between members of the defined reference protein set. After appropriate updating of the old and filtering of the obsolete protein identifiers, the acquired PPI data were normalized to and combined at the UniProt protein identifier level. We analyzed the reconstructed network to discuss whether the revealed role of proteins based on their position in the interactome topology is supported by the currently available knowledge about their function. In addition, based on the verified scale-free structure of the PPI network in human [1,30], we predict the number of connections of the unrepresented proteins and provide a novel perspective about the presently “missing” part of the interactome.

## Methods

### Protein and PPI datasets

#### The UniProtKB/Swiss-Prot manually reviewed human “complete” proteome

From UniProtKB, the knowledgebase of the Universal Protein (UniProt) resource [24], we downloaded the tab-delimited files of: (a) the entire set of human UniProt identifiers, and (b) the manually reviewed human “complete” proteome. The latter contained 20,242 UniProt identifiers in the Dec 14 2011 release of UniProtKB downloaded on Jan 23 2012. The two tab-delimited files included all default columns augmented by the cross-references with the EMBL nucleotide, the NCBI nucleotide and the Entrez Gene databases. The text file indicating the correspondence of the secondary to the respective primary UniProt identifier(s) was downloaded too.

#### The Human Protein Reference Database (HPRD)

HPRD is a manually curated reference database for human protein information [25]. In this study, we used only its binary PPI dataset, which is provided in the form of interactions between HPRD identifiers. From the total 19651 HPRD identifiers in the HPRD version 9, downloaded on Jan 23 2012, 9673 were involved in at least one of the 39204 PPIs reported as binary interactions. Only the primary one-to-one correspondence of the HPRD identifiers to nucleotide sequence identifiers was considered. Any necessary updating or conversion of the nucleotide sequence identifiers to other molecular levels of biological information (i.e. gene or protein level) was carried out through cross-reference with current versions of the relevant databases.

#### IntAct

IntAct, a main partner of the International Molecular Exchange (IMEx) Consortium [10], is a repository of molecular interaction data for multiple organisms [26]. In the single file supplied by IntAct for external use, including interaction information from all species, PPIs are provided mainly at the UniProt protein identifier level. From the Jan 3, 2012 release downloaded on Jan 30, 2012, only the non - “spoke” PPIs between two human protein identifiers were retained, as the label “spoke” characterizes the PPIs originated from protein complex expansion.

#### The Molecular INTeraction database (MINT)

Similarly to IntAct, MINT [27] is a repository of literature-curated PPIs from multiple organisms and an IMEx consortium partner with PPI information provided mainly at the UniProt protein identifier level. The binary PPI file for human used in the present study was downloaded on Jan 30, 2012 (release date: Dec 8, 2011).

#### Database of Interacting Proteins (DIP)

DIP [28] is also a collection of experimentally supported protein interactions from multiple organisms and among the first partners of the IMEx consortium. In the downloaded on Jan 30, 2012 PPI file for human (release date: Oct 27 2011), PPIs are provided as interactions between DIP identifiers. The latter are corresponded mainly to UniProt protein identifier(s) and most to NCBI nucleotide RefSeq identifier(s), too.

#### The Biological General Repository for Interaction Datasets (BioGRID)

BioGRID [29] is the most recently initiated among the five source PPI databases used in this study, currently participating in the IMEx consortium as an affiliate member. The PPI file for human was downloaded from the BioGRID web site on Jan 30, 2012 (release 3.1.84 tab2 file). PPIs are provided as interactions between BioGRID identifiers, which are in one to one correspondence to Entrez Gene identifiers (GeneID). BioGRID provides extensive information about the experimental method and the nature, i.e. low- or high- throughput, of the experimental set-up used for any PPI detection; however, it does neither make a distinction between binary interaction and protein complex data nor provide a relevant filtering criterion. To avoid including PPI data expanded from protein complexes, we opted to keep (a) all physical associations identified in low-throughput setups and (b) from the physical associations detected only in high-throughput experiments, those derived from any of “protein complementation assay (PCA)”, “reconstituted complex”, “protein-peptide”, “FRET”, “two-hybrid” or “co-crystal structure” methods. Genetic interactions provided in BioGRID were *de facto* filtered out.

### PPI data mining

Direct PPIs with both interactors belonging to the set of the 20,242 primary UniProt identifiers included in the manually reviewed human “complete” proteome were mined from: (a) the binary PPI dataset of HPRD, (b) all PPIs of IntAct not characterized with the term “spoke” in the “expansion” field, (c) the binary PPI dataset of MINT, (d) the DIP dataset, which is provided as containing only binary manually reviewed PPIs, and (e) all physical associations in BioGRID detected in at least one low-throughput experiment or by any of the detection methods mentioned above, if identified only in high-throughput setups.

### Protein identifier normalization

Normalization of the protein identifiers to the UniProt identifier level was required for: (a) HPRD, since it reports the interactors at the nucleotide sequence level, (b) BioGRID, which reports the interactors at the gene level and (c) few cases of IntAct, MINT and DIP, for which other than the default UniProt identifier has been used.

### Source PPI dataset uploading

To upload, store and handle the five PPI datasets and integrate them into the final reconstructed PPI network, the Microsoft SQL Server (MSSQL) 2008 Developer Edition platform equipped with SQL Server Integration Services (SSIS) was used under the University of Patras academic license. The source PPI dataset uploading was organized in a set of SSIS modules executed at the server side. Each module involves a series of subtasks for the filtering and updating of certain data from the source PPI dataset, along with a large number of checks to monitor and handle exceptions, avoiding thus the contamination of the final database with erroneous or ill-formatted data. Additional file 1 shows the workflow for the IntAct uploading sub-module.

The first subtask of the filtering and updating algorithm involves the extraction of the interactions between human protein identifiers. In sequence, the main interactor identifiers are retained for each PPI. For IntAct, MINT and DIP, the interactors are expected to be represented by a UniProtKB accession number. If the relevant format is not recovered from the algorithm for any of the two interactors, then the non-UniProt interactor identifier is compared against a maintained interactor identifier dictionary. If matched to a dictionary entry and identified as active, the non-UniProt interactor identifier is replaced by the corresponding primary UniProt identifier. If it has become obsolete or cannot be assigned to a UniProtKB accession number, it is removed from the finally uploaded dataset along with all associated PPIs. If active, all isoform UniProt protein identifiers are replaced by their primary UniProt identifier(s). Any remaining non-UniProt interactor identifiers are stored in a separate table, for the curator to appropriately update the interactor identifier dictionary, so that the “patching” process is completed in a second iteration. In HPRD, the interactor identifier dictionary is used to update the nucleotide sequence identifiers to their currently active entries. Notably, among the 9673 HPRD identifiers involved in PPIs, 119 were identified to correspond to obsolete nucleotide sequence identifiers, 4 corresponded to non protein-coding RNAs, while 16 were replaced by new nucleotide sequence identifiers; due to this updating, in three cases, two HPRD identifiers were assigned to the same nucleotide sequence identifier. In BioGRID, all interactors were identified by an active Entrez GeneID, thus no updating was necessary. For the PPIs remaining after the interactor identifier patching step, the algorithm inspects the identifier of the supporting publication(s). If no publication is provided, the PPI is removed from the uploaded dataset. If a non-PubMed publication identifier is provided, this is patched based on an in-memory maintained dictionary as described for the interactor identifiers in the previous step. The utilized interactor identifier dictionary was created based on information recovered from the online UniProt converter and the online versions of all relevant databases on February 2, 2012. The Digital Object Identifier (DOI) numbers and IMEx reference identifiers were assigned to their PubMed publication identifiers based on an online converter and the online version of MINT, respectively. After uploading IntAct, MINT and DIP, their PPI data were further processed based on information from UniProtKB to include only interactions between two active primary UniProt identifiers in the human manually reviewed “complete” proteome.

### Gene functional classification analysis

Gene functional classification analysis was carried out using the DAVID Bioinformatics Resources version 6.7 [31,32] by combining all available gene annotation categorizations.

### Identification of network characteristics

The identification of the reconstructed PPI network characteristics was carried out using the relevant “Network Analysis” tool of the open source network visualization and analysis software Cytoscape - version 2.8 [33].

## Results and discussion

### Reconstructing the human protein interactome based on a well-defined set of protein nodes

The novelty of our approach regarding the PPI data integration from major literature-curated source PPI datasets compared to existing meta-databases was the *a priori* definition of the set of nodes of the human protein interactome considering the UniProtKB manually reviewed human “complete” proteome as a robust, well-defined reference set. Thus, instead of merging PPI information for any protein identifier stored in the source databases, the latter were selectively mined for PPIs exclusively between members of the as above defined reference human protein set.

For proper normalization of the source PPI datasets to the UniProt identifier level, it was also important to consider the continuous updating of biological information, since it can lead to changes in the annotation of protein identifiers and in their associations at other molecular levels. Thus, we proceeded to a careful updating of the old and filtering of the obsolete protein identifiers in the source datasets based on the current knowledge about gene annotation. UniProtKB and its cross-references with major resources at the nucleotide sequence and gene levels of molecular information (i.e. NCBI, Entrez Gene and EMBL databases) provided a valuable reference for the appropriate normalization of HPRD and BioGRID identifiers to the UniProt level, and of a small fraction of IntAct, MINT and DIP protein entries that were not provided at the default UniProt level. It is noted that during this conversion to the UniProt level, 1920 BioGRID identifiers reported as human were found to correspond to non-human UniProt identifiers (data not shown), leading thus to the exclusion of their PPIs from the final integrated PPI network.

In the normalized HPRD, IntAct, MINT, DIP and BioGRID files, only the PPIs between two active primary UniProt identifiers in the manually reviewed human “complete” proteome were retained. These datasets were combined keeping one record for each included PPI. A last source of PPI redundancy in the normalized datasets that was eliminated, concerns the double reporting of an interaction using opposite sequence of the two interactors. In some cases, such duplications may have been intentionally included by the curator of a source PPI dataset to report the experimentally supported sequence of the interactors; this type of duplications were encountered in IntAct and MINT. In most cases, however, they were just a product of the protein identifier conversions at the various stages of the PPI dataset uploading and formatting and had to be eliminated at the integration stage.

The final integrated PPI dataset will be referred to as the PICKLE (Protein InteraCtion KnowLedge BasE) dataset. Table 1 shows the number of (a) the direct PPIs in the PICKLE and the normalized source PPI datasets, (b) the UniProt identifiers in the manually reviewed human “complete” proteome covered by each of them, and (c) the publications providing experimental evidence for the PPIs. As expected, the integrated PICKLE dataset is much larger than any of the individual source datasets with respect to the number of PPIs, of the protein interactors and of the supporting publications, verifying the value of PPI resource integration.

**Table 1:** The size of the reconstructed direct PPI network for the manually reviewed human “complete” proteome

	**Number of UniProt Identifiers**^**(1)**^	**Number of Direct PPIs**^**(2) **^**(x)**	**Number of Supporting Publications (y)**	**Mean Number of PPIs per Publication (x/y)**
**UniProtKB manually reviewed human “complete” proteome**	20242	N/A	N/A	N/A
**HPRD**	9303	37152	19267	1.93
**IntAct**	6666	19425	1598	12.15
**MINT**	6102	16147	2398	6.73
**DIP**	1795	2609	1180	2.21
**BioGRID**	9265	42647	13818	3.08
**PICKLE**	**11827**	**75965**	**26689**	**2.85**

The size of the reconstructed network is compared to the size of the normalized to the UniProt identifier level source PPI datasets for the defined reference protein set. N/A: not applicable.

^(1)^members of the UniProtKB manually reviewed human “complete” proteome.

^(2)^between members of the UniProtKB manually reviewed human “complete” proteome.

Reconstructing the PPI network in this global structured way:

•we resolve the issue of potential protein identifier and consequently PPI redundancy in the network originating from the combination of records of multiple databases reporting at different levels of biological information;

•we determine which protein nodes of the manually reviewed human “complete” proteome remain with no direct PPIs (“orphan” proteins) and discuss this fact in the context of the current information about these proteins;

•we comment on the proteins represented in the interactome with a high number of PPIs with respect to the importance of their function within the entire network;

•we consider the human interactome in its entirety, commenting on its future expansion to the maximum potential format in the context of the expected scale-free structure, a fundamental feature of PPI networks [30,34]. Consequently, the interactome reconstructed in the presented way can only grow in edges (PPIs) between the defined set of protein nodes, while keeping its scale-free form. In this global context, we can argue for the expected number of interactions for the “orphan” protein nodes and for the type of their interactors, suggesting a novel perspective for the currently “missing” part of the network, as it is discussed in the following sections.

### The reconstructed interactome covers nearly 60% of the manually reviewed human “complete” proteome

Out of the 20,242 UniProt identifiers in the manually reviewed human “complete” proteome, 11827 (58.4%) were found to have a total number of 75965 direct interactions (Table 1). Gene functional classification analysis (see Methods section) of the proteins currently included in the reconstructed interactome compared to the “orphan” ones did not indicate any functional annotations that could differentiate the one group from the other. Thus, the presently “orphan” proteins are not associated with any apparent functional or subcellular location characteristics that could “hinder” them from binding with other proteins.

### Dataset integration augments the overall network mainly with additional interactions for largely overlapping sets of proteins

**Figure 1:**
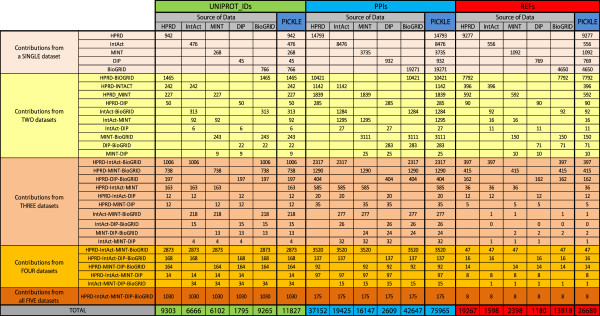
Source of data in the integrated PICKLE PPI dataset.

**Figure 2:**
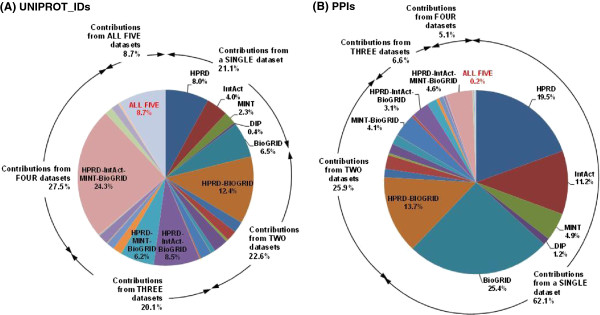
**The fractions of PICKLE UniProt identifiers (A) and PPIs (B) contributed from combinations of source datasets.** The common contributions for the nodes and the edges of the integrated PPI network from all five source datasets constitute 8.7% and 0.2% of the total, respectively. Only the values of the larger than 3% fractions are shown with the exception of the unique contributions from each individual source dataset, for which all fractions are indicated.

**Figure 3:**
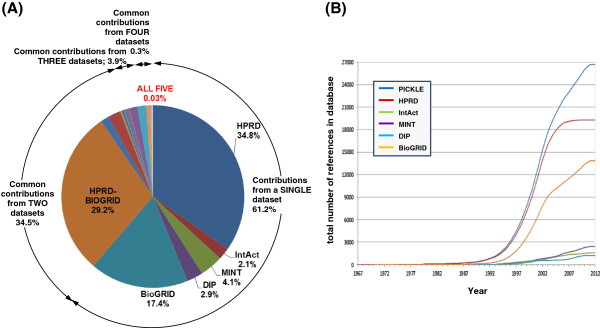
**The PICKLE reference fraction contributed from source datasets (A) and the reference incorporation rate in the datasets (B).** Only 8 common references between the five datasets were identified, confirming that they incorporate knowledge from different studies. In **(A)**, only the values of the larger than 3% fractions are shown with the exception of the unique contributions from each individual source dataset, for which all fractions are indicated.

**Figure 4:**
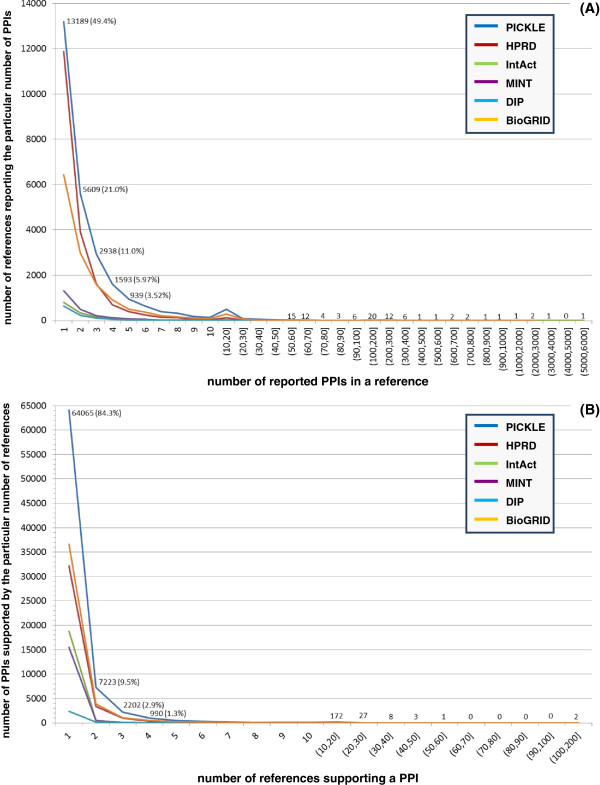
Distribution of PPIs per reference (A) and supporting references per PPI (B) in PICKLE.

HPRD and BioGRID are the main contributors of the overall human PPI network, comprising, respectively, 78.7% and 78.3% of its UniProt identifiers, and 48.9% and 56.1% of its PPIs (Table 1 and Figures 1, 2 and 3). Moreover, exclusion of the information from HPRD and BioGRID wοuld, respectively, decrease the overall network by 20.4% and 18.9% in proteins and 33.2% and 39.1% in PPIs. These characteristics can be partially justified by the number of references used by each of these two databases, constituting 72.2% (HPRD) and 51.8% (BioGRID) of the total number of supporting references. In addition, HPRD is one of the first literature-curated databases, having though a decline in the rate of reference (and thus PPI) incorporation after 2005 (Figure 3B). BioGRID is currently the fastest growing, having also incorporated a significant part of the HPRD PPI network at the time of its creation [11,29]. This information complements the observed much higher curation overlap between HPRD and BioGRID compared to the other pairs of source PPI datasets discussed by Turinsky et al. in [12]. On the other hand, IntAct corresponds to the largest ratio of PPIs per number of references, i.e. 12.1, followed by MINT, i.e. 6.7 (Table 1), indicating that a major fraction of their datasets originates from references of high-throughput PPI experiments. Notably, the reconstructed human protein interactome is mainly supported by small-scale studies (Figure 4A); 91% of the references supporting the PICKLE PPI dataset refer to a maximum of five PPIs, and only 51 publications report more than 100 PPIs. In this aspect, PICKLE follows the characteristics of HPRD, currently the main contributor of references to the overall dataset. It is worth mentioning that 84% of the 75965 PPIs in the human interactome are supported by only one reference (Figure 4B) and just 42 PPIs by more than 20 (Additional file 2). Considering that the degree of confidence of a given PPI increases with the number of independent supporting references [35], it is evident that, apart from exploiting existing models for PPI assessment [36], further targeted experimentation is required for validating the majority of the PPI data.

A noteworthy observation of our work, revealing an interesting aspect of the literature-supported PPI data collections, is that the fraction of protein nodes that each source dataset uniquely contributes to the integrated network is much smaller than the corresponding fraction for the PPIs, even for the largest HPRD and BioGRID datasets (Figures 1 and 2). The PPI diversity between the source datasets has been discussed earlier [e.g. 10, 12] and mainly attributed to the fact that the various databases incorporate knowledge from different publications. This was recently presented for the IMEx Consortium member databases [10] and validated in the present study from the substantially small number, i.e. eight, of common references between the five employed datasets (Figure 1). Furthermore, Turinsky et al. [12] showed that the source databases exploit different curation criteria even for the shared publications. Thus, it is striking that, despite the heterogeneous text mining and data curation methods used by the various databases, the integration of multiple source PPI datasets augments mainly the interactome with different PPIs for essentially the same part of the manually reviewed human “complete” proteome.

This observation suggests that the knowledge about direct PPIs that is available in the literature and can be promptly identified through existing text mining algorithms refers mainly to the fraction, i.e. approximately 60%, of the manually reviewed human “complete” proteome already incorporated in the interactome, while evidence for PPIs for the rest 40% cannot be easily spotted. In this context, as PPI information from all high-throughput experiments has been included in at least one of the source datasets, there are two possibilities for the “orphan” proteins: either there is currently no available PPI information in the literature, or, if existing, it should concern reports of targeted small-scale functional experiments. From this kind of reports, protein interactions can be indirectly deduced, requiring thus advanced directed text mining algorithms. Furthermore, there is a higher probability for such experiments to refer to PPIs occurring under specialized and/or highly transient or rare physiological conditions, while this type of interactions cannot be easily identified in high-throughput experiments. These implied direct interaction characteristics for the “orphan” proteins support a peripheral role for most of them within the topology of the PPI network. In this context, the actual determination of the “orphan” proteins may assist in directed literature mining to extract potentially existing relevant PPI information from currently unexploited reports or promote further experimentation to verify the argument.

### The proteins with a high number of interactions are involved in essential biological processes

**Figure 5:**
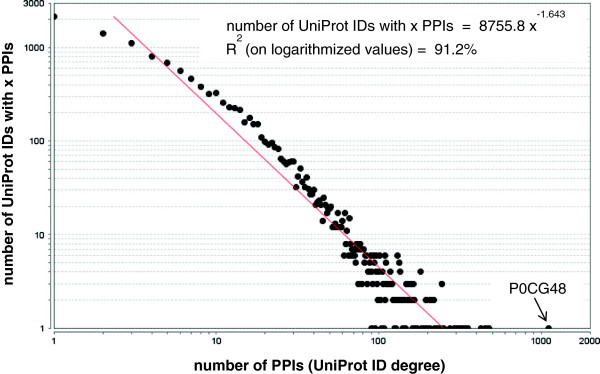
**The distribution of direct interactions for the UniProt identifiers in the PICKLE network.** The red line indicates the power-law fit (logarithmic scale); the related equation and R^2^ correlation are also shown. UniProt identifier P0CG48 (Polyubiquitin, UBC) is identified with the largest number of interactions, i.e. 1112; note the deviation of this UniProt identifier degree from the power-law fit.

Analysis of the integrated human PPI network characteristics indicated that 11577 out of the 11827 UniProt identifiers are connected in one component. The remaining 250 proteins are currently in separate components of up to four nodes, among which 114 homodimers and 46 heterodimers (Table 2). The vastest functional categories for these proteins as indicated by gene functional classification analysis concerned 107 glycoproteins, 64 of which are homodimers and 89 signal peptides, among which 65 glycoproteins; 68 of the signal peptides, including 39 glycoproteins, are associated with extracellular matrix. While the network diameter, i.e. the greatest distance between two protein nodes, was determined equal to 12, the characteristic path length is 3.69. This feature along with the equal to 1 radius and the high value of shortest paths metric (i.e. 95%) indicates a well-connected network, despite its low density (i.e. 0.001) (Table 2). The distribution of PPIs per protein, i.e. protein degree, indicated 53% of the proteins as having up to five interactions (Figure 5), while 16 UniProt identifiers had more than 300 PPIs each (Table 3). This pattern is consistent with the relevant “network biology” theory supported by Barabasi [30,37], according to which the human PPI network is expected to follow a scale-free structure with few protein hubs and the majority of the protein nodes having a small number of interactions. Indeed, even though it is currently incomplete and many interactions are still in need of verification, the reconstructed human protein interactome correlates well with the power law (Figure 5), implying that the degree distribution of the current PPI network already suggests the role of most proteins as high-, middle- or low- degree nodes.

**Table 2:** The characteristics of the integrated PPI network

**Network characteristic**^**(1)**^	**Value**^**(2)**^
Number of Nodes	11827
Isolated Nodes (homodimers)	114
Connected components	174 (i.e.: 1 cluster of 11577 nodes, 114 homodimers, 46 heterodimers,13 isolated of 3 or 4 nodes)
Number of self-loops	2715 (i.e.: 2601 nodes having interactions with other proteins as well, and 114 isolated homodimers)
Network radius	1
Network diameter	12
Characteristic Path Length	3.691
Average Number of Neighbors	12.387
Shortest Paths	95%
Clustering Coefficient	0.127
Network Density	0.001
Network Centralization	0.093
Network Heterogeneity	2.193

^(1)^Detailed description for every characteristic can be found in http://med.bioinf.mpi-inf.mpg.de/netanalyzer/help/2.6.1/index.html*.*

^(2)^Determined using the network analysis tool of Cytoscape (2.8.2).

**Table 3:** The 16 UniProt identifiers with more than 300 interactions in the integrated PPI network

**UniProt Identifier**	**UniProt Entry Name**	**Gene Symbol**	**Protein Name(s) (based on UniProt Naming Convention)**	**No of PPIs (Degree)**
P0CG48	UBC_HUMAN	UBC	Polyubiquitin-C [Cleaved into: Ubiquitin]	1112
P04637	P53_HUMAN	TP53	Cellular tumor antigen p53 (Antigen NY-CO-13) (Phosphoprotein p53) (Tumor suppressor p53)	476
P63104	1433Z_HUMAN	YWHAZ	14-3-3 protein zeta/delta (Protein kinase C inhibitor protein 1) (KCIP-1)	471
P01106	MYC_HUMAN	MYC	Myc proto-oncogene protein (Class E basic helix-loop-helix protein 39) (bHLHe39) (Proto-oncogene c-Myc) (Transcription factor p64)	453
Q9Y4K3	TRAF6_HUMAN	TRAF6	TNF receptor-associated factor 6 (EC 6.3.2.-) (E3 ubiquitin-protein ligase TRAF6) (Interleukin-1 signal transducer) (RING finger protein 85)	424
Q13547	HDAC1_HUMAN	HDAC1	Histone deacetylase 1 (HD1) (EC 3.5.1.98)	353
P12931	SRC_HUMAN	SRC	Proto-oncogene tyrosine-protein kinase Src (EC 2.7.10.2) (Proto-oncogene c-Src) (pp60c-src) (p60-Src)	351
P62993	GRB2_HUMAN	GRB2	Growth factor receptor-bound protein 2 (Adapter protein GRB2) (Protein Ash) (SH2/SH3 adapter GRB2)	341
Q14164	IKKE_HUMAN	IKBKE	Inhibitor of nuclear factor kappa-B kinase subunit epsilon (I-kappa-B kinase epsilon) (IKK-E) (IKK-epsilon) (IkBKE) (EC 2.7.11.10) (Inducible I kappa-B kinase) (IKK-i)	338
P61981	1433G_HUMAN	YWHAG	14-3-3 protein gamma (Protein kinase C inhibitor protein 1) (KCIP-1) [Cleaved into: 14-3-3 protein gamma, N-terminally processed]	335
Q09472	EP300_HUMAN	EP300	Histone acetyltransferase p300 (p300 HAT) (EC 2.3.1.48) (E1A-associated protein p300)	331
Q9UQL6	HDAC5_HUMAN	HDAC5	Histone deacetylase 5 (HD5) (EC 3.5.1.98) (Antigen NY-CO-9)	324
P00533	EGFR_HUMAN	EGFR	Epidermal growth factor receptor (EC 2.7.10.1) (Proto-oncogene c-ErbB-1) (Receptor tyrosine-protein kinase erbB-1)	313
P03372	ESR1_HUMAN	ESR1	Estrogen receptor (ER) (ER-alpha) (Estradiol receptor) (Nuclear receptor subfamily 3 group A member 1)	312
P27348	1433T_HUMAN	YWHAQ	14-3-3 protein theta (14-3-3 protein T-cell) (14-3-3 protein tau) (Protein HS1)	311
Q9Y6K9	NEMO_HUMAN	IKBKG	NF-kappa-B essential modulator (NEMO) (FIP-3) (IkB kinase-associated protein 1) (IKKAP1) (Inhibitor of nuclear factor kappa-B kinase subunit gamma) (I-kappa-B kinase subunit gamma) (IKK-gamma) (IKKG) (IkB kinase subunit gamma) (NF-kappa-B essential modifier)	304

The sixteen proteins determined with more than 300 PPIs (Table 3) are mainly implicated in the regulation of apoptosis (10 proteins), the MAP kinase signalling pathway (6 proteins) and the cell cycle (7 proteins). A full list of the most significant protein ontology clusters for these high-degree proteins is shown in Additional file 3. Notably, eight of them have been associated with pathways in cancer, while subsets of nine are involved in transcription regulation, covalent chromatin modification or the ubiquitin-like modifier (ubl) conjugation pathway. This information indicates that the observed central role of these proteins within the topology of the PPI network is not a mere result of them being extensively studied, i.e. “study bias”, but correlates well with the current knowledge about their function, as it has also been suggested earlier for the cancer-associated proteins [38,39]. An additional fact which counter argues the “study bias” for these proteins is that, apart from various targeted small-scale experiments, many of their direct interactions have also been detected in independent high-throughput setups. For example, at least 54 interactions of the cellular tumor antigen p53 [8,40], 257 interactions of the 14-3-3 protein zeta/delta [41], 212 interactions of the Myc proto-oncogene protein [42] and 48 interactions of the TNF receptor-associated factor 6 [43] have been identified in high-throughput studies.

### Polyubiquitin: a hub to be discussed

Polyubiquitin (UniProt identifier: P0CG48, UBC) was the protein identified with the largest number of interactions in the reconstructed network. It interacts with more than a thousand, i.e.: 1112, members of the manually reviewed human “complete” proteome, while the second ranked high-degree node, i.e.: TP53 (UniProt identifier: P04637), has 476 interactions. Notably, this much larger number of interactions for polyubiquitin compared to the other protein hubs deviates from the scale-free network structure, assigning a centralized role to a single protein (Figure 5). Querying the PICKLE dataset, we identified HPRD, IntAct, MINT, DIP and BioGRID reporting, respectively, 19 (2 unique), 5 (0 unique), 143 (48 unique), 53 (15 unique) and 1423 (909 unique) polyubiquitin PPIs. Without exhausting our search regarding polyubiquitin PPI supporting publications, we detected that our integrated dataset contains interactions from studies investigating polyubiquitin function in the context of protein degradation (e.g. [44]). Polyubiquitin can be covalently linked to a protein through an isopeptide bond and mark it for degradation at the proteasome. However, it is questionable whether this one-sided polyubiquitin action on a protein should be included in the interactome or should be considered in the post-translational modification (PTM) network [45,46]. The latter could explain why, apart from BioGRID, the other source databases used in this work have considered a limited number of polyubiquitin PPIs. In the context of the non-directional PPI network, the existence of an interaction link from one protein to another directly implies a link in the opposite direction, too. Consequently, the absence of a protein and thus its interactions will affect its neighbours and add a certain stress to the network, the extent of which depends on the network structure and dynamics. In the case of unidirectional polyubiquitination of a protein for leading it to degradation, the absence of the protein will neither affect polyubiquitin nor exert a stress to the rest of the polyubiquitin substrates. Thus, this type of actions of a protein on another should be considered as a separate category than the non-directional protein-protein interactions and modelled differently for their role in cell physiology dynamics. On the other hand, the monoubiquitination of proteins for regulatory purposes (e.g. [47]) fits into the notion of the non-directional PPI network. However, even in this case, it is questionable whether ubiquitin itself or rather the ubiquitinated proteins should be included as nodes of the network. In this context, the incorporation of ubiquitin PPIs in the interactome should be cautiously curated. Accordingly, this argument is also relevant to other proteins involved in interactions of similar type, like the small ubiquitin-related modifiers (SUMO1-4) and neddylin (NEDD8) engaged in the sumoylation and neddylation reactions, respectively.

### The bulk of the proteins currently absent from the network should have up to four interactions

As shown, the reconstructed human protein interactome follows the scale-free structure with a very good correlation (Figure 5). The part of the network that contributes to the decrease in the correlation coefficient refers to the proteins with up to four interactions. The difference between the data and the power-law curve for a nearly perfect fit is calculated to be about 8300 UniProt identifiers, with more than 6500 of them corresponding to degree equal to 1. Consequently, with 8415 UniProt identifiers not currently included in the interactome (“orphan” proteins), it could be speculated that the vast majority of them should have up to four interactions with nodes in the same degree group. This anticipated network structure implies that the core of the human protein interactome has essentially been revealed and could provide a reasonable explanation for the current lack of PPI information for about 40% of the human proteome, agreeing with a specialized “peripheral” role for most of these “orphan” proteins. Indeed, with most of them expected to have a single PPI, and in general no more than four, with similarly not well-connected proteins, the probability of them being involved in specialized physiological conditions is high. This speculation further corroborates with the fact that interactions for these proteins cannot be easily confirmed in PPI identification experiments, as discussed in section C.

## Conclusions

We have obtained a normalized and clean from outdated protein identifier annotations integrated set of direct PPIs referring to the well-defined UniProtKB manually reviewed human “complete” proteome. We suggest that this PPI network with the involvement of approximately 60% of the “complete” proteome represents the core of the human protein interactome. Based on a global view of the way in which the current network will have to expand to its maximum potential in accordance with the scale-free theory, we provide a novel perspective for suggesting its currently “missing” part. We envisage that the proteins not yet identified in direct PPI assays may participate in specialized biological functions interacting with a limited number of other not well-connected proteins. Now determined, this set of “orphan” proteins may trigger targeted text mining efforts or appropriately designed functional experiments for the identification of any relevant PPIs. In effect, we suggest that this reconstructed human interactome already provides a useful tool for generating valuable working hypotheses for the investigation of important biological processes and molecular functions in the context of biomedical research and applications.

## Competing interests

The authors declare that they have no competing interests.

## Authors’ contributions

NKM conceived and coordinated the study; MIK, AT and NKM participated in the design of the study; MIK, KT and NKM selected the source datasets; MIK supervised and KT created the dictionaries for the protein and publication identifier updating; MIK and ET designed and validated the data uploading process; ET developed the data uploading modules; MIK and NKM analyzed the reconstructed network; MIK drafted and NKM finalized the manuscript. All authors read and approved the final manuscript.

## Supplementary Material

Additional file 1 The uploading process flowchart for the IntAct PPI dataset using MS-SQL Integration Services (print screen shot). Click here for file

Additional file 2 List of the 42 PPIs supported by 20 or more references in the reconstructed network. Click here for file

Additional file 3 **The major annotation clusters of the 16 UniProt identifiers with the largest number of PPIs.** The UniProt identifier list is provided in Table 3. The clusters were determined by the functional annotation software DAVID using all relevant gene annotation categorizations. Click here for file

## References

[B1] Barabási AL Gulbahce N Loscalzo J Network medicine: a network-based approach to human disease Nat Rev Genet 2011 7 56 68 10.1038/nrg2918 21164525 PMC3140052

[B2] Sharma A Gulbahce N Pevzner S Menche J Ladenvall C Folkersen L Eriksson P Orho-Melander M Barabási AL Network based analysis of genome wide association data provides novel candidate genes for lipid and lipoprotein traits Mol Cell Proteomics 2013 Epub ahead of print 10.1074/mcp.M112.024851 PMC3820950 23882023

[B3] Li S Armstrong CM Bertin N Ge H Milstein S Boxem M Vidalain PO Han JD Chesneau A Hao T Goldberg DS Li N Martinez M Rual JF Lamesch P Xu L Tewari M Wong SL Zhang LV Berriz GF Jacotot L Vaglio P Reboul J Hirozane-Kishikawa T Li Q Gabel HW Elewa A Baumgartner B Rose DJ Yu H Bosak S Sequerra R Fraser A Mango SE Saxton WM Strome S Van Den Heuvel S Piano F Vandenhaute J Sardet C Gerstein M Doucette-Stamm L Gunsalus KC Harper JW Cusick ME Roth FP Hill DE Vidal M A map of the interactome network of the metazoan *C. elegans* Sci 2004 7 540 543 10.1126/science.1091403 PMC1698949 14704431

[B4] Giot L Bader JS Brouwer C Chaudhuri A Kuang B Li Y Hao YL Ooi CE Godwin B Vitols E Vijayadamodar G Pochart P Machineni H Welsh M Kong Y Zerhusen B Malcolm R Varrone Z Collis A Minto M Burgess S McDaniel L Stimpson E Spriggs F Williams J Neurath K Ioime N Agee M Voss E Furtak K Renzulli R Aanensen N Carrolla S Bickelhaupt E Lazovatsky Y DaSilva A Zhong J Stanyon CA Finley RL Jr White KP Braverman M Jarvie T Gold S Leach M Knight J Shimkets RA McKenna MP Chant J Rothberg JM A protein interaction map of *Drosophila melanogaster* Sci 2003 7 1727 1736 10.1126/science.1090289 14605208

[B5] Gavin AC Bösche M Krause R Grandi P Marzioch M Bauer A Schultz J Rick JM Michon AM Cruciat CM Remor M Höfert C Schelder M Brajenovic M Ruffner H Merino A Klein K Hudak M Dickson D Rudi T Gnau V Bauch A Bastuck S Huhse B Leutwein C Heurtier MA Copley RR Edelmann A Querfurth E Rybin V Drewes G Raida M Bouwmeester T Bork P Seraphin B Kuster B Neubauer G Superti-Furga G Functional organization of the yeast proteome by systematic analysis of protein complexes Nat 2002 7 141 147 10.1038/415141a 11805826

[B6] Ito T Ota K Kubota H Yamaguchi Y Chiba T Sakuraba K Yoshida M Roles for the two-hybrid system in exploration of the yeast protein interactome Mol Cell Proteomics 2002 7 561 566 10.1074/mcp.R200005-MCP200 12376571

[B7] Uetz P Giot L Cagney G Mansfield TA Judson RS Knight JR Lockshon D Narayan V Srinivasan M Pochart P Qureshi-Emili A Li Y Godwin B Conover D Kalbfleisch T Vijayadamodar G Yang M Johnston M Fields S Rothberg JM A comprehensive analysis of protein-protein interactions in *Saccharomyces cerevisiae* Nat 2000 7 623 627 10.1038/35001009 10688190

[B8] Stelzl U Worm U Lalowski M Haenig C Brembeck FH Goehler H Stroedicke M Zenkner M Schoenherr A Koeppen S Timm J Mintzlaff S Abraham C Bock N Kietzmann S Goedde A Toksöz E Droege A Krobitsch S Korn B Birchmeier W Lehrach H Wanker EE A human protein-protein interaction network: a resource for annotating the proteome Cell 2005 7 957 968 10.1016/j.cell.2005.08.029 16169070

[B9] Rual JF Venkatesan K Hao T Hirozane-Kishikawa T Dricot A Li N Berriz GF Gibbons FD Dreze M Ayivi-Guedehoussou N Klitgord N Simon C Boxem M Milstein S Rosenberg J Goldberg DS Zhang LV Wong SL Franklin G Li S Albala JS Lim J Fraughton C Llamosas E Cevik S Bex C Lamesch P Sikorski RS Vandenhaute J Zoghbi HY Smolyar A Bosak S Sequerra R Doucette-Stamm L Cusick ME Hill DE Roth FP Vidal M Towards a proteome-scale map of the human protein-protein interaction network Nat 2005 7 1173 1178 10.1038/nature04209 16189514

[B10] Orchard S Kerrien S Abbani S Aranda B Bhate J Bidwell S Bridge A Briganti L Brinkman Fiona SL Cesareni G Chatr-aryamontri A Chautard E Chen C Dumousseau M Goll J Hancock Robert EW Hannick LI Jurisica I Khadake J Lynn DJ Mahadevan U Perfetto L Raghunath A Ricard-Blum S Roechert B Salwinski L Stümpflen V Tyers M Uetz P Xenarios I Hermjakob H Protein interaction data curation: the International Molecular Exchange (IMEx) consortium Nat Methods 2012 7 345 350 10.1038/nmeth.1931 22453911 PMC3703241

[B11] Klingström T Plewczynski D Protein-protein interaction and pathway databases, a graphical review Brief Bioinform 2011 7 702 713 Epub 2010 Sep 17 10.1093/bib/bbq064 20851835

[B12] Turinsky AL Razick S Turner B Donaldson IM Wodak SJ Literature curation of protein interactions: measuring agreement across major public databases Database (Oxford) 2010 7 2010:baq026 10.1093/database/baq026 PMC3011985 21183497

[B13] Cusick ME Yu H Smolyar A Venkatesan K Carvunis AR Simonis N Rual JF Borick H Braun P Dreze M Vandenhaute J Galli M Yazaki J Hill DE Ecker JR Roth FP Vidal M Literature-curated protein interaction datasets Nat Methods 2009 7 39 46 10.1038/nmeth.1284 19116613 PMC2683745

[B14] Mathivanan S Periaswamy B Gandhi TK Kandasamy K Suresh S Mohmood R Ramachandra YL Pandey A An evaluation of human protein-protein interaction data in the public domain BMC Bioinforma 2006 7 Suppl 5 S19 10.1186/1471-2105-7-S5-S19 PMC1764475 17254303

[B15] Kamburov A Stelzl U Lehrach H Herwig R The ConsensusPathDB interaction database: 2013 update Nucleic Acids Res 2013 7 Database issue D793 D800 23143270 10.1093/nar/gks1055 PMC3531102

[B16] Razick S Magklaras G Donaldson IM iRefIndex: a consolidated protein interaction database with provenance BMC Bioinforma 2008 7 405 10.1186/1471-2105-9-405 PMC2573892 18823568

[B17] Prieto C De Las RJ APID: Agile Protein Interaction DataAnalyzer Nucleic Acids Res 2006 7 W298 W302 10.1093/nar/gkl128 16845013 PMC1538863

[B18] Schaefer MH Fontaine JF Vinayagam A Porras P Wanker EE Andrade-Navarro MA HIPPIE: Integrating protein interaction networks with experiment based quality scores PLoS One 2012 7 e31826 10.1371/journal.pone.0031826 22348130 PMC3279424

[B19] Jayapandian M Chapman A Tarcea VG Yu C Elkiss A Ianni A Liu B Nandi A Santos C Andrews P Athey B States D Jagadish HV Michigan molecular interactions r2: from interacting proteins to pathways Nucleic Acids Res 2009 7 Database issue D642 D646 18978014 10.1093/nar/gkn722 PMC2686565

[B20] Das J Yu H HINT: High-quality protein interactomes and their applications in understanding human disease BMC Syst Biol 2012 7 92 10.1186/1752-0509-6-92 22846459 PMC3483187

[B21] Cowley MJ Pinese M Kassahn KS Waddell N Pearson JV Grimmond SM Biankin AV Hautaniemi S Wu J PINA v2.0: mining interactome modules Nucleic Acids Res 2012 7 Database issue D862 D865 22067443 10.1093/nar/gkr967 PMC3244997

[B22] Chaurasia G Malhotra S Russ J Schnoegl S Hänig C Wanker EE Futschik ME UniHI 4: new tools for query, analysis and visualization of the human protein-protein interactome Nucleic Acids Res 2009 7 Database issue D657 D660 18984619 10.1093/nar/gkn841 PMC2686569

[B23] Cerami EG Gross BE Demir E Rodchenkov I Babur O Anwar N Schultz N Bader GD Sander C Pathway Commons, a web resource for biological pathway data Nucleic Acids Res 2011 7 Database issue D685 D690 21071392 10.1093/nar/gkq1039 PMC3013659

[B24] The UniProt Consortium Reorganizing the protein space at the Universal Protein Resource (UniProt) Nucleic Acids Res 2012 7 D71 D75 22102590 10.1093/nar/gkr981 PMC3245120

[B25] Keshava Prasad TS Goel R Kandasamy K Keerthikumar S Kumar S Mathivanan S Telikicherla D Raju R Shafreen B Venugopal A Balakrishnan L Marimuthu A Banerjee S Somanathan DS Sebastian A Rani S Ray S Harrys Kishore CJ Kanth S Ahmed M Kashyap MK Mohmood R Ramachandra YL Krishna V Rahiman BA Mohan S Ranganathan P Ramabadran S Chaerkady R Pandey A Human Protein Reference Database--2009 update Nucleic Acids Res 2009 7 Database issue D767 D772 Epub 2008 Nov 6 18988627 10.1093/nar/gkn892 PMC2686490

[B26] Kerrien S Aranda B Breuza L Bridge A Broackes-Carter F Chen C Duesbury M Dumousseau M Feuermann M Hinz U Jandrasits C Jimenez RC Khadake J Mahadevan U Masson P Pedruzzi I Pfeiffenberger E Porras P Raghunath A Roechert B Orchard S Hermjakob H The IntAct molecular interaction database in 2012 Nucleic Acids Res 2011 7 Database issue D841 D846 Epub 2011 Nov 24 22121220 10.1093/nar/gkr1088 PMC3245075

[B27] Licata L Briganti L Peluso D Perfetto L Iannuccelli M Galeota E Sacco F Palma A Nardozza AP Santonico E Castagnoli L Cesareni G MINT, the molecular interaction database: 2012 update Nucleic Acids Res 2011 7 Database issue D857 D861 Epub 2011 Nov 16 22096227 10.1093/nar/gkr930 PMC3244991

[B28] Salwinski L Miller CS Smith AJ Pettit FK Bowie JU Eisenberg D The Database of Interacting Proteins: 2004 update Nucleic Acids Res 2004 7 Database issue D449 D451 14681454 10.1093/nar/gkh086 PMC308820

[B29] Stark C Breitkreutz BJ Reguly T Boucher L Breitkreutz A Tyers M Biogrid: A General Repository for Interaction Datasets Nucleic Acids Res 2006 7 D535 D539 10.1093/nar/gkj109 16381927 PMC1347471

[B30] Barabasi A-L Oltvai ZN Network biology: understanding the cell's functional organization Nat Rev Genet 2004 7 101 113 10.1038/nrg1272 14735121

[B31] Huang DW Sherman BT Lempicki RA Systematic and integrative analysis of large gene lists using DAVID Bioinformatics Resources Nature Protoc 2009 7 44 57 10.1038/nprot.2008.211 19131956

[B32] Huang DW Sherman BT Lempicki RA Bioinformatics enrichment tools: paths toward the comprehensive functional analysis of large gene lists Nucleic Acids Res 2009 7 1 13 10.1093/nar/gkn923 19033363 PMC2615629

[B33] Smoot M Ono K Ruscheinski J Wang P-L Ideker T Cytoscape 2.8: new features for data integration and network visualization Bioinform 2011 7 431 432 10.1093/bioinformatics/btq675 PMC3031041 21149340

[B34] Zhu Y Zhang XF Dai DQ Wu MY Identifying spurious interactions and predicting missing interactions in the protein-protein interaction networks via a generative network model IEEE/ACM Trans Comput Biol Bioinform 2013 7 219 225 23702559 10.1109/TCBB.2012.164

[B35] Yu J Finley RL Jr Combining multiple positive training sets to generate confidence scores for protein-protein interactions Bioinform 2009 7 105 111 10.1093/bioinformatics/btn597 PMC2638943 19010802

[B36] McDowall MD Scott MS Barton GJ PIPs: human protein-protein interaction prediction database Nucleic Acids Res 2009 7 Database issue D651 D656 18988626 10.1093/nar/gkn870 PMC2686497

[B37] Yook SH Oltvai ZN Barabási AL Functional and topological characterization of protein interaction networks Proteomics 2004 7 928 942 10.1002/pmic.200300636 15048975

[B38] Jonsson PF Bates PA Global topological features of cancer proteins in the human interactome Bioinform 2006 7 2291 2297 10.1093/bioinformatics/btl390 PMC1865486 16844706

[B39] Ghersi D Singh M Disentangling function from topology to infer the network properties of disease genes BMC Syst Biol 2013 7 5 10.1186/1752-0509-7-5 23324116 PMC3614482

[B40] Vinayagam A Stelzl U Foulle R Plassmann S Zenkner M Timm J Assmus HE Andrade-Navarro MA Wanker EE A directed protein interaction network for investigating intracellular signal transduction Sci Signal 2011 7 rs8 10.1126/scisignal.2001699 21900206

[B41] Meek SE Lane WS Piwnica-Worms H Comprehensive proteomic analysis of interphase and mitotic 14-3-3-binding proteins J Biol Chem 2004 7 32046 32054 10.1074/jbc.M403044200 15161933

[B42] Koch HB Zhang R Verdoodt B Bailey A Zhang CD Yates JR 3rd Menssen A Hermeking H Large-scale identification of c-MYC-associated proteins using a combined TAP/MudPIT approach Cell Cycle 2007 7 205 217 10.4161/cc.6.2.3742 17314511

[B43] Bouwmeester T Bauch A Ruffner H Angrand PO Bergamini G Croughton K Cruciat C Eberhard D Gagneur J Ghidelli S Hopf C Huhse B Mangano R Michon AM Schirle M Schlegl J Schwab M Stein MA Bauer A Casari G Drewes G Gavin AC Jackson DB Joberty G Neubauer G Rick J Kuster B Superti-Furga G A physical and functional map of the human TNF-alpha/NF-kappa B signal transduction pathway Nat Cell Biol 2004 7 97 105 10.1038/ncb1086 14743216

[B44] Venancio TM Balaji S Iyer LM Aravind L Reconstructing the ubiquitin network: cross-talk with other systems and identification of novel functions Genome Biol 2009 7 R33 10.1186/gb-2009-10-3-r33 19331687 PMC2691004

[B45] Du Y Xu N Lu M Li T hUbiquitome: a database of experimentally verified ubiquitination cascades in humans Database (Oxford) 2001 7 bar055 10.1093/database/bar055 PMC3228279 22134927

[B46] Matsumoto M Hatakeyama S Oyamada K Oda Y Nishimura T Nakayama KI Large-scale analysis of the human ubiquitin-related proteome Proteomics 2005 7 4145 4151 10.1002/pmic.200401280 16196087

[B47] Koutelou E Sato S Tomomori-Sato C Florens L Swanson SK Washburn MP Kokkinaki M Conaway RC Conaway JW Moschonas NK Neuralized-like 1 (Neurl1) targeted to the plasma membrane by N-myristoylation regulates the Notch ligand Jagged1 J Biol Chem 2008 7 3846 3853 18077452 10.1074/jbc.M706974200

