# Supplementary material for: Reconstruction of the experimentally supported human protein interactome: what can we learn?
Source: BMC Syst Biol. 2013 Oct 2;7:96. doi: 10.1186/1752-0509-7-96 (PMC4015887; doi:10.1186/1752-0509-7-96)
Supplement: Additional file 1 — The uploading process flowchart for the IntAct PPI dataset using MS-SQL Integration Services (print screen shot). [file 1752-0509-7-96-S1.pptx]

## Slide 1
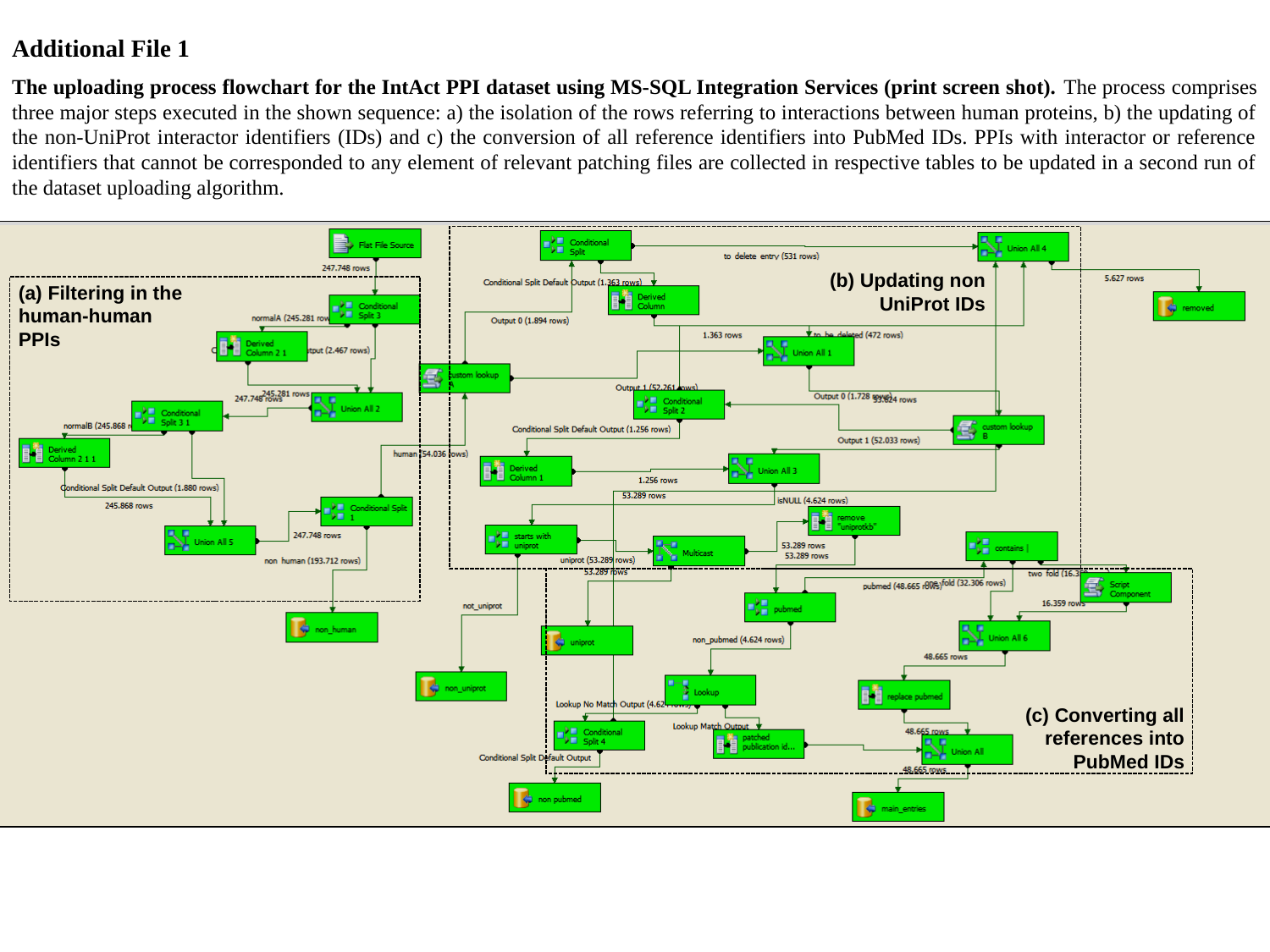

Additional File 1
The uploading process flowchart for the IntAct PPI dataset using MS-SQL Integration Services (print screen shot). The process comprises three major steps executed in the shown sequence: a) the isolation of the rows referring to interactions between human proteins, b) the updating of the non-UniProt interactor identifiers (IDs) and c) the conversion of all reference identifiers into PubMed IDs. PPIs with interactor or reference identifiers that cannot be corresponded to any element of relevant patching files are collected in respective tables to be updated in a second run of the dataset uploading algorithm.
(b) Updating non UniProt IDs
(a) Filtering in the human-human PPIs
(c) Converting all references into PubMed IDs
