# Supplementary material for: Reconstruction of the experimentally supported human protein interactome: what can we learn?
Source: BMC Syst Biol. 2013 Oct 2;7:96. doi: 10.1186/1752-0509-7-96 (PMC4015887; doi:10.1186/1752-0509-7-96)
Supplement: Additional file 2 — List of the 42 PPIs supported by 20 or more references in the reconstructed network. [file 1752-0509-7-96-S2.pdf]

Additional File 2

List of the 42 PPIs supported by 20 or more references in the reconstructed network.

INTERACTOR A	INTERACTOR B	Number of supporting references
P01100	P05412	20
P0CG48	Q16236	21
P62877	Q13616	21
P11802	P24385	21
P12004	P38936	21
P63208	Q13616	21
Q14145	Q16236	22
P04637	P04637	22
P0CG48	Q16665	22
Q09028	Q13547	23
O15360	Q00597	24
Q13547	Q92769	24
P12830	P35222	24
P08047	Q13547	25
P06400	Q01094	25
O14920	Q9Y6K9	25
P06730	Q13541	25
P0CG48	Q9Y6K9	25
P06400	Q13547	25
Q13485	Q15796	26
P51587	Q06609	26
P49815	Q92574	26
P00533	P62993	27
P42345	Q8N122	27
P25963	Q04206	29
O60563	P50750	30
P04637	Q09472	30
Q13547	Q96ST3	30
P62993	Q07889	32
P22681	P62993	33
P01112	P04049	34
P0CG48	Q00987	34
P01106	P61244	36
P0CG48	Q9Y4K3	36
P29353	P62993	38
P00533	P22681	39
P40337	Q16665	41
O15287	O15360	41
P38398	Q99728	42
P00533	P0CG48	51
P04637	P0CG48	112
P04637	Q00987	146
