# Supplementary material for: Reconstruction of the experimentally supported human protein interactome: what can we learn?
Source: BMC Syst Biol. 2013 Oct 2;7:96. doi: 10.1186/1752-0509-7-96 (PMC4015887; doi:10.1186/1752-0509-7-96)
Supplement: Additional file 3 — The major annotation clusters of the 16 UniProt identifiers with the largest number of PPIs. The UniProt identifier list is provided in Table 3. The clusters were determined by the functional annotation software DAVID using all relevant gene annotation categorizations. [file 1752-0509-7-96-S3.pdf]

Additional File 3

The major annotation clusters of the 16 UniProt IDs with the largest number of PPIs. The UniProt ID list is provided in Table 4. The clusters were determined by the functional annotation software DAVID using all relevant gene annotation categorizations.

Annotation Cluster No	Cluster Enrichment Score	Number of Genes involved	List of Genes involved	Gene Functional Annotations Included in this Cluster (number of genes involved)
1	5.36	12	TRAF6<br>EGFR<br>ESR1<br>HDAC1<br>IKBKG<br>TP53<br>YWHAZ<br>UBC<br>MYC<br>SRC<br>HDAC5<br>EP300	GO:0042981~regulation of apoptosis (10)<br>GO:0043067~regulation of programmed cell death (10)<br>GO:0010941~regulation of cell death (10)<br>GO:0043066~negative regulation of apoptosis (8)<br>GO:0043069~negative regulation of programmed cell death (8)<br>GO:0060548~negative regulation of cell death (8)<br>GO:0006916~anti-apoptosis (6)<br>isopeptide bond (6)<br>GO:0031328~positive regulation of cellular biosynthetic process (8)<br>GO:0009891~positive regulation of biosynthetic process (8)<br>GO:0043065~positive regulation of apoptosis (6)<br>GO:0043068~positive regulation of programmed cell death (6)<br>GO:0010942~positive regulation of cell death (6)<br>GO:0043085~positive regulation of catalytic activity(6)<br>GO:0022402~cell cycle process (4)<br>GO:0051240~positive regulation of multicellular organismal process (3)
2	4.27	7	TRAF6<br>EGFR<br>HDAC1<br>IKBKG<br>TP53<br>UBC<br>MYC	isopeptide bond (6)<br>cross-link:Glycyl lysine isopeptide (Lys-Gly) (interchain with G-Cter in SUMO) (4)<br>hsa05222:Small cell lung cancer (4)
3	3.35	7	TRAF6<br>EGFR<br>GRB2<br>IKBKG<br>TP53<br>MYC<br>HDAC1	hsa05220:Chronic myeloid leukemia (5)<br>hsa04010:MAPK signaling pathway (6)<br>hsa05221:Acute myeloid leukemia (3)

4	3.17	11	EP300<br>HDAC1<br>TP53<br>YWHAG<br>YWHAQ<br>YWHAZ<br>MYC<br>EGFR<br>GRB2<br>TRAF6<br>SRC	hsa04110:Cell cycle (7)<br>hsa04722:Neurotrophin signaling pathway (6)<br>site:Interaction with phosphoserine on interacting protein (3)<br>IPR000308:14-3-3 protein (3)<br>SM00101:14_3_3 (3)<br>PIRSF000868:14-3-3 protein (3)<br>PIRSF000868:14-3-3 (3)<br>GO:0019904~protein domain specific binding (5)<br>GO:0034613~cellular protein localization (5)<br>GO:0070727~cellular macromolecule localization (5)<br>GO:0006605~protein targeting (4)<br>GO:0006886~intracellular protein transport (4)<br>GO:0008104~protein localization (5)<br>hsa04114:Oocyte meiosis (3)<br>GO:0046907~intracellular transport (4)<br>GO:0015031~protein transport (4)<br>GO:0045184~establishment of protein localization (4)<br>GO:0000267~cell fraction (4)
5	3.12	7	EP300<br>EGFR<br>ESR1<br>GRB2<br>HDAC5<br>MYC<br>SRC	GO:0010033~response to organic substance (7)<br>h_her2Pathway:Role of ERBB2 in Signal Transduction and Oncology (4)<br>GO:0009725~response to hormone stimulus (5)<br>GO:0009719~response to endogenous stimulus (6)<br>h_pelp1Pathway:Pelp1 Modulation of Estrogen Receptor Activity (3)<br>GO:0048545~response to steroid hormone stimulus (3)
6	2.84	10	EP300<br>TRAF6<br>EGFR<br>HDAC5<br>IKBKG<br>TP53<br>UBC<br>MYC<br>HDAC1<br>GRAB2	GO:0044093~positive regulation of molecular function(8)<br>isopeptide bond (6)<br>hsa05215:Prostate cancer (5)<br>GO:0043085~positive regulation of catalytic activity(6)<br>hsa04010:MAPK signaling pathway (6)<br>cross-link:Glycyl lysine isopeptide (Lys-Gly) (interchain with G-Cter in ubiquitin) (4)<br>hsa05212:Pancreatic cancer (3)<br>GO:0046982~protein heterodimerization activity (3)<br>GO:0042592~homeostatic process (4)<br>GO:0001501~skeletal system development (3)<br>disease mutation (4)<br>GO:0046983~protein dimerization activity (3)
7	2.67	12	EP300<br>TRAF6<br>EGFR<br>HDAC5<br>IKBKG<br>TP53<br>UBC<br>MYC<br>GRB2<br>SRC<br>ESR1<br>HDAC1	GO:0044093~positive regulation of molecular function(8)<br>hsa05200:Pathways in cancer (8)<br>hsa05220:Chronic myeloid leukemia (5)<br>GO:0010033~response to organic substance (7)<br>hsa05213:Endometrial cancer (4)<br>hsa05210:Colorectal cancer (4)<br>hsa04012:ErbB signaling pathway (4)<br>GO:0008283~cell proliferation (4)<br>Proto-oncogene (3)<br>GO:0006461~protein complex assembly (4)<br>GO:0070271~protein complex biogenesis (4)<br>GO:0065003~macromolecular complex assembly (4)<br>GO:0043933~macromolecular complex subunit organization (4)<br>alternative splicing (7)<br>splice variant (7)

8	2.47	14	EP300<br>GRB2<br>HDAC1<br>HDAC5<br>TP53<br>YWHAG<br>YWHAQ<br>YWHAZ<br>UBC<br>MYC<br>ESR1<br>TRAF6<br>IKBKG<br>EGFR	GO:0044093~positive regulation of molecular function (8)<br>GO:0031328~positive regulation of cellular biosynthetic process (8)<br>GO:0009891~positive regulation of biosynthetic process (8)<br>GO:0010604~positive regulation of macromolecule metabolic process (8)<br>GO:0010628~positive regulation of gene expression (7)<br>acetylation (10)<br>GO:0051173~positive regulation of nitrogen compound metabolic process (7)<br>GO:0010557~positive regulation of macromolecule biosynthetic process (7)<br>GO:0060284~regulation of cell development (5)<br>GO:0045941~positive regulation of transcription (6)<br>GO:0048742~regulation of skeletal muscle fiber development (3)<br>GO:0045935~positive regulation of nucleobase, nucleoside, nucleotide and nucleic acid metabolic process (6)<br>GO:0048641~regulation of skeletal muscle tissue development (3)<br>GO:0051153~regulation of striated muscle cell sifferentiation (5)<br>GO:0045944~positive regulation of transcription from RNA polymerase II promoter (3)<br>GO:0051147~regulation of muscle cell differentiation (6)<br>GO:0010605~negative regulation of macromolecule metabolic process (3)<br>GO:0016202~regulation of striated muscle tissue development (3)<br>GO:0048634~regulation of muscle development (5)<br>GO:0045893~positive regulation of transcription, DNA-dependent (5)<br>GO:0051254~positive regulation of RNA metabolic process (3)<br>GO:0010552~positive regulation of specific transcription from RNA polymerase II promoter (6)<br>GO:0005654~nucleoplasm (5)<br>GO:0010629~negative regulation of gene expression (5)<br>GO:0008134~transcription factor binding (5)<br>GO:0044451~nucleoplasm part (7)<br>transcription regulation (9)<br>GO:0045449~regulation of transcription (7)<br>Transcription (5)<br>GO:0006915~apoptosis (5)<br>GO:0012501~programmed cell death (3)<br>GO:0043193~positive regulation of gene-specific transcription (3)<br>GO:0010551~regulation of specific transcription from RNA polymerase II promoter (7)<br>GO:0030528~transcription regulator activity<br>compositionally biased region:Poly-Gln (3)<br>GO:0045892~negative regulation of transcription, DNA-dependent (4)<br>GO:0008219~cell death (5)<br>GO:0051253~negative regulation of RNA metabolic process (4)<br>GO:0016265~death (5)

				GO:0006357~regulation of transcription from RNA polymerase II promoter (5)<br>GO:0016570~histone modification (3)<br>GO:0016569~covalent chromatin modification (3)<br>nucleus (9)<br>GO:0006355~regulation of transcription, DNA-dependent (7)<br>GO:0007049~cell cycle (5)<br>GO:0032583~regulation of gene-specific transcription(3)<br>GO:0051252~regulation of RNA metabolic process (3)<br>GO:0007005~mitochondrion organization (7)<br>GO:0016481~negative regulation of transcription (3)<br>GO:0016604~nuclear body (4)<br>GO:0051276~chromosome organization (3)<br>GO:0031981~nuclear lumen (4)<br>GO:0045934~negative regulation of nucleobase, nucleoside, nucleotide and nucleic acid metabolic process (6)<br>GO:0051172~negative regulation of nitrogen compound metabolic process (4)<br>GO:0006350~transcription (4)<br>GO:0010558~negative regulation of macromolecule biosynthetic process (7)<br>GO:0003700~transcription factor activity (4)<br>GO:0031327~negative regulation of cellular biosynthetic process (5)<br>GO:0042493~response to drug (3)<br>GO:0045596~negative regulation of cell differentiation (3)<br>GO:0009890~negative regulation of biosynthetic process (4)<br>GO:0043565~sequence-specific DNA binding (4)<br>GO:0000122~negative regulation of transcription from RNA polymerase II promoter (3)<br>GO:0070013~intracellular organelle lumen (6)<br>GO:0016568~chromatin modification (3)<br>GO:0003677~DNA binding (7)<br>GO:0043233~organelle lumen (6)<br>GO:0031974~membrane-enclosed lumen (6)<br>GO:0042592~homeostatic process (4)<br>hsa04310:Wnt signaling pathway (3)<br>GO:0006325~chromatin organization (3)<br>hsa05016:Huntington's disease (3)<br>GO:0043232~intracellular non-membrane-bounded organelle (6)<br>GO:0043228~non-membrane-bounded organelle (6)
9	2.44	12	EP300<br>TRAF6<br>EGFR<br>ESR1<br>HDAC1<br>HDAC5<br>IKBKG<br>TP53<br>UBC<br>MYC<br>SRC<br>YWHAZ	ubl conjugation (9)<br>GO:0044093~positive regulation of molecular function (8)<br>GO:0031328~positive regulation of cellular biosynthetic process (8)<br>GO:0009891~positive regulation of biosynthetic process (8)<br>GO:0010604~positive regulation of macromolecule metabolic process (8)<br>GO:0010628~positive regulation of gene expression (7)<br>GO:0043065~positive regulation of apoptosis (6)<br>GO:0043068~positive regulation of programmed cell death (6)<br>GO:0010942~positive regulation of cell death (6)<br>GO:0043085~positive regulation of catalytic activity (6)<br>zinc finger (4)<br>GO:0045941~positive regulation of transcription (6)<br>GO:0006917~induction of apoptosis (5)

				GO:0012502~induction of programmed cell death (5)<br>GO:0045935~positive regulation of nucleobase, nucleoside, nucleotide and nucleic acid metabolic process (6)<br>hsa05222:Small cell lung cancer (4)<br>GO:0005654~nucleoplasm (6)<br>GO:0006915~apoptosis (5)<br>GO:0012501~programmed cell death (5)<br>GO:0030528~transcription regulator activity (7)<br>GO:0008219~cell death (5)<br>GO:0016265~death (5)<br>nucleus (9)<br>GO:0007049~cell cycle (5)<br>99.NF-kB_activation (3)<br>GO:0031981~nuclear lumen (6)<br>zinc (6)<br>GO:0022402~cell cycle process (4)<br>GO:0048584~positive regulation of response to stimulus (3)<br>GO:0070013~intracellular organelle lumen (6)<br>GO:0043233~organelle lumen (6)<br>zinc-finger (5)<br>GO:0031974~membrane-enclosed lumen (6)<br>GO:0048598~embryonic morphogenesis (3)<br>GO:0042592~homeostatic process (4)<br>GO:0043009~chordate embryonic development (3)<br>GO:0009792~embryonic development ending in birth or egg hatching (3)<br>GO:0040008~regulation of growth (3)<br>metal-binding (6)<br>GO:0008270~zinc ion binding (6)<br>GO:0043232~intracellular non-membrane-bounded organelle (6)<br>GO:0043228~non-membrane-bounded organelle (6)<br>GO:0044265~cellular macromolecule catabolic process (3)<br>GO:0046914~transition metal ion binding (6)<br>GO:0009057~macromolecule catabolic process (3)<br>GO:0046872~metal ion binding (6)<br>GO:0043169~cation binding (6)<br>GO:0043167~ion binding (6)
10	2.17	5	HDAC1<br>HDAC5<br>TP53<br>YWHAG<br>UBC	GO:0060284~regulation of cell development (5)<br>GO:0051960~regulation of nervous system development (3)<br>GO:0043086~negative regulation of catalytic activity (3)<br>GO:0044092~negative regulation of molecular function (3)
11	2.14	5	TRAF6<br>EGFR<br>HDAC5<br>TP53<br>YWHAZ	GO:0001775~cell activation (5)<br>GO:0045321~leukocyte activation (4)<br>GO:0002521~leukocyte differentiation (3)<br>GO:0030097~hemopoiesis (3)<br>GO:0048534~hemopoietic or lymphoid organ development (3)<br>GO:0002520~immune system development (3)

12	2.1	12	TRAF6<br>EGFR<br>IKBKG<br>TP53<br>UBC<br>MYC<br>GRB2<br>HDAC1<br>EP300<br>ESR1<br>SRC<br>YWHAG	hsa05215:Prostate cancer (5)<br>GO:0043085~positive regulation of catalytic activity (6)<br>hsa05213:Endometrial cancer (4)<br>hsa04010:MAPK signaling pathway (6)<br>hsa05210:Colorectal cancer (4)<br>GO:0045786~negative regulation of cell cycle (3)<br>hsa05219:Bladder cancer (3)<br>GO:0051726~regulation of cell cycle (4)<br>GO:0003690~double-stranded DNA binding (3)<br>hsa05223:Non-small cell lung cancer (3)<br>GO:0007049~cell cycle (5)<br>hsa05214:Glioma (3)<br>GO:0008283~cell proliferation (4)<br>GO:0043566~structure-specific DNA binding (3)<br>GO:0007346~regulation of mitotic cell cycle (3)<br>GO:0051345~positive regulation of hydrolase activity (3)<br>GO:0009314~response to radiation (3)<br>GO:0019899~enzyme binding (4)<br>GO:0022402~cell cycle process (4)<br>GO:0001501~skeletal system development (3)<br>GO:0051336~regulation of hydrolase activity (3)<br>GO:0042127~regulation of cell proliferation (4)<br>GO:0009628~response to abiotic stimulus (3)<br>GO:0008284~positive regulation of cell proliferation (3)<br>glycoprotein (4)
13	2.06	11	TRAF6<br>EGFR<br>ESR1<br>GRB2<br>IKBKG<br>TP53<br>YWHAQ<br>SRC<br>YWHAG<br>MYC<br>IKBKE	GO:0007242~intracellular signaling cascade (8)<br>hsa04010:MAPK signaling pathway (6)<br>GO:0007243~protein kinase cascade (5)<br>GO:0045859~regulation of protein kinase activity (4)<br>GO:0043549~regulation of kinase activity (4)<br>GO:0032147~activation of protein kinase activity (3)<br>GO:0051338~regulation of transferase activity (4)<br>hsa05120:Epithelial cell signaling in Helicobacter pylori infection (3)<br>GO:0042325~regulation of phosphorylation (4)<br>kinase (4)<br>GO:0051174~regulation of phosphorus metabolic process(4)<br>GO:0019220~regulation of phosphate metabolic process (4)<br>GO:0045860positive regulation of protein kinase activity(3)<br>GO:0033674~positive regulation of kinase activity (3)<br>GO:0051347~positive regulation of transferase activity (3)<br>hsa04144:Endocytosis (3)<br>GO:0007166~cell surface receptor linked signal transduction (5)
14	2.02	6	TRAF6<br>EGFR<br>GRB2<br>IKBKG<br>SRC<br>MYC	GO:0007173~epidermal growth factor receptor signaling pathway (3)<br>GO:0007243~protein kinase cascade (5)<br>hsa04012:ErbB signaling pathway (4)<br>h_cblPathway:CBL mediated ligand-induced downregulation of EGF receptors (3)<br>h_spryPathway:Sprouty regulation of tyrosine kinase signals (3)<br>h_At1rPathway:Angiotensin II mediated activation of JNK Pathway via Pyk2 dependent signaling (3)<br>h_erkPathway:Erk1/Erk2 Mapk Signaling pathway (3)<br>hsa04540:Gap junction (3)<br>GO:0007169~transmembrane receptor protein tyrosine kinase signaling pathway (3)<br>hsa04912:GnRH signaling pathway (3)<br>GO:0007167~enzyme linked receptor protein signaling

				pathway (3)<br>hsa04510:Focal adhesion (3)<br>GO:0007166~cell surface receptor linked signal transduction (5)
15	1.89	5	TRAF6<br>IKBKE<br>IKBKG<br>TP53<br>YWHAZ	GO:0006955~immune response (5)<br>hsa04622:RIG-I-like receptor signaling pathway (3)<br>hsa04620:Toll-like receptor signaling pathway (3)
16	1.81	3	EGFR<br>YWHAG<br>UBC	GO:0050804~regulation of synaptic transmission (3)<br>GO:0051969~regulation of transmission of nerve impulse(3)<br>GO:0031644~regulation of neurological system process (3)<br>GO:0044057~regulation of system process (3)
17	1.62	6	TRAF6<br>EGFR<br>ESR1<br>IKBKE<br>UBC<br>SRC	GO:0010647~positive regulation of cell communication (6)<br>GO:0044459~plasma membrane part (3)<br>GO:0005886~plasma membrane (3)
